# Supplementary material for: Prevalence of Rome IV Pediatric Diagnostic Questionnaire-Assessed Disorder of Gut–Brain Interaction, Psychopathological Comorbidities and Consumption of Ultra-Processed Food in Pediatric Anorexia Nervosa
Source: Nutrients. 2024 Mar 13;16(6):817. doi: 10.3390/nu16060817 (PMC10975836; doi:10.3390/nu16060817)
Supplement: Supplementary file 1 [file nutrients-16-00817-s001.zip › nutrients-2875949-supplementary.pdf]

DGBIs according to the R4PDQ

Functional Dyspepsia (FD)

Functional dyspepsia is diagnosed if child qualifies for postprandial distress syndrome or epigastric pain syndrome or both

Postprandial Distress Syndrome (PDS):

- Fullness "4 days a month" or more often, OR
- Satiation "4 days a month" or more often, AND
- Duration of upper abdominal pain or discomphort is "2 months" or longer.

Epigastric Pain Syndrome (EPS):

- Upper abdominal pain "4 days a month" or more often, OR heartburn "4 days a month" or more often, AND
- Duration of upper abdominal pain or discomfort is "2 months" or longer, AND
- Not related to a bowel movement, AND
- Not associated with change in stool form or frequency.

Irritable Bowel Syndrome (IBS)

- Abdominal pain "4 days a month" or more often, AND
- Abdominal pain is "2 months" or longer, AND
- Not exclusively associated with eating, AND
- For girls, non exclusively associated with menses, AND
- For those who use laxatives, symptoms persist with their use, AND
- At last one of the following:
    - Pain or discomfort occurring just before or after evacuation.
    - Stools are softer, creamier, or more watery than usual
    - Stools are harder or more granular than usual
    - Bowel movement is more frequent than usual
    - Bowel movement is less frequent than usual.

Functional Constipation (FC)

Must include two or more of the following:

- Two or fewer stools per week, OR
- Hard or Very hard stools OR Painful stool, OR
- Passage of very large stools, OR
- Stool retention "1 time a week" or more often, OR

- History of large fecal mass in rectum, OR
- Soiling “Once a week” or more often
- If child meets criteria for IBS, pain should improve with laxative use

Adolescent Rumination Syndrome

- Food come back up “Several times a week” or “Every day” AND
- Episodes occur shortly after eating, AND
- Episodes do not occur during sleep, AND
- Episodes are not accompained by nausea or vomiting.
